# Well‐Being in the Nation: A Living Library of Measures to Drive Multi‐Sector Population Health Improvement and Address Social Determinants

**DOI:** 10.1111/1468-0009.12477

**Published:** 2020-09-01

**Authors:** SOMAVA SAHA, BRUCE B. COHEN, JULIA NAGY, MARIANNE E. McPHERSON, ROBERT PHILLIPS

**Affiliations:** ^1^ Well‐being and Equity (WE) in the World; ^2^ Harvard Medical School; ^3^ Well Being In the Nation Network; ^4^ Institute for Healthcare Improvement; ^5^ 100 Million Healthier Lives; ^6^ National Committee on Vital and Health Statistics; ^7^ American Board of Family Medicine

## Abstract

**Policy Points**

- Well‐being In the Nation (WIN) offers the first parsimonious set of vetted common measures to improve population health and social determinants across sectors at local, state, and national levels and is driven by what communities need to improve health, well‐being, and equity.
- The WIN measures were codesigned with more than 100 communities, federal agencies, and national organizations across sectors, in alignment with the National Committee on Vital and Health Statistics, the Foundations for Evidence‐Based Policymaking Act, and Healthy People 2030. WIN offers a process for a collaborative learning measurement system to drive a learning health and well‐being system across sectors at the community, state, and national levels.
- The WIN development process identified critical gaps and opportunities in equitable community‐level data infrastructure, interoperability, and protections that could be used to inform the Federal Data Strategy.

The past five years has seen a substantial increase in initiatives and activities that foster community‐level multisector collaboration to advance population health. The rise in awareness of how social determinants drive health outcomes, advanced by the County Health Rankings & Roadmaps, the National Academies of Science, Engineering and Medicine, the All‐In, the Practical Playbook, 100 Million Healthier Lives, the Robert Wood Johnson Foundation's Culture of Health initiative, and many others, have moved communities to invest in multisector collaboration to address social needs and the social determinants of health.

While there seems to be general agreement that multisector collaboration is needed to improve population health and address social determinants, the availability of common multisector measurement and data infrastructure that meet the needs of diverse sectors to support such collaboration remains in its infancy.^1^ A study of 237 multisector partnerships found that more than half of them engaged 10 or more sectors, with public health and health care organizations most frequently in the lead role.^2^ These partnerships have struggled in part because they lack common measures across sectors that can be easily used to drive improvement at the local (subcounty) level. Numerous studies point to the need for more timely data at smaller geographic levels to more effectively focus programs and resources.^3^, ^4^, ^5^, ^6^ The absence of a mechanism for multisector collaboration at both the national and the community level to identify common measures and promote learning across sectors, as well as the growing awareness that it is not possible to address social needs or social determinants without such collaboration, contributed to the demand for publicly available measures that support community collaborations to assess population health, address social determinants, and improve well‐being and health equity.

## The Development of the NCVHS Framework

In its advisory role to the US Department of Health and Human Services (HHS), the National Committee on Vital and Health Statistics (NCVHS) studied data gaps identified by stakeholders engaged in community health assessment and improvement work to help address these challenges. During this process, the committee became aware of the loss of several federal data and community measurement tools over the past five years, including the Health Indicators Warehouse, which undermined communities’ ability to improve health and well‐being outcomes^7^.

From 2011 to 2016, the committee convened a series of hearings and meetings on data‐driven approaches to improving the nation's health^8^. The Committee identified and documented the essential role of community‐level data in measurably improving population health and well‐being. As a result of feedback from data users and stakeholders, in 2016 NCVHS commissioned an environmental scan of existing multisector approaches to measure and assess health.^9^ Based on this and input from more than 90 thought leaders, researchers, and stakeholders at the final meeting, the committee released two significant documents: recommendations to the HHS secretary and a measurement framework for community health and well‐being grounded in the social determinants of health.^10^, ^11^ The framework consisted of 10 broad domains—community vitality, demographics, economy, education, environment, food and agriculture, health, housing, public safety, and transportation—with 30 subdomains of measures identified as important during the committee's multiyear process. The process and principles that guided the framework's development were documented in the committee's final two reports on the topic.^11^, ^12^

NCVHS achieved its goal of forming a foundational approach to support the measurement of health and well‐being at the local, state, and national levels with the release of the framework in January 2017. Subsequently, the committee sought a nongovernmental entity to oversee the framework's development, maturation, pilot, implementation, and refinement in collaboration with federal, state, and local governmental and nongovernmental organizations. The resulting 100 Million Healthier Lives initiative, a collaboration of partners across sectors convened by the Institute for Healthcare Improvement, accepted responsibility for this process in coordination with NCVHS.

## Methods

### Principles of the Approach

The NCVHS Measurement Framework led to two key recommendations that served as guiding principles for this effort.^11^ Specifically, NCVHS recommended that a measurement framework (1) be *flexible* enough to meet distinct local needs with a focus on subcounty and community‐level data and multisector measures and (2) provide a *parsimonious* set of multisector core measures to guide federal and state policy and resource allocation and enable communities to compare one another and share best practices in 10 domains.

The stewardship group that guided this process made two additional recommendations: (1) achieve a *balance* between standard, widely used, measures and “developmental” measures (those measures that may be promising and might be useful later in understanding population and community health, well‐being, and equity); and (2) ensure that the framework is *informed by experience* based on the measurement's development, implementation, and field testing at the local, state, and national levels.

The 100 Million Healthier Lives team used the following principles to guide its approach in accordance with this charge and its commitment to an equitable process that would lead to measures useful to communities:

**1. Codesign**. The team identified who would be using these measures, from the community level to the national level, and brought these implementers together with measurement experts to identify and select measures. The implementers were more than 100 organizations, from community residents to policymakers to major organizations in many sectors (health care, public health, business, economy, transportation, housing, etc.) who could bring their perspective and unique needs to the table as well as help align related measurement efforts across sectors. To date, Well‐Being In the Nation (WIN) is the only collaboration of this scale across sectors that identifies measures for population and community health together with the priority of improving equity and outcomes at the community (subcounty) level.
**2. Continual alignment**. WIN repeatedly conducted landscape analyses of the field during its 18‐month development cycle to both engage new measurement efforts and integrate emerging findings. It aligned intentionally with Healthy People 2030, 500 Cities, County Health Rankings & Roadmaps, City Health Dashboard, National Academies, *US News & World Report*, HOPE measures, and many others, through conversation and metrics alignment and by bringing team members into the development processes.
**3. Continual testing in the field with those who would use it**. As the key concepts, measure domains, and measures were identified, they were continually tested in local communities and across sectors. This allowed WIN to respond to the needs of different stakeholders in practical ways and helped achieve an understanding of the different kinds of measures needed by different stakeholders.
**4. Flexibility and balance in measure selection, adaptation, and use**. To meet the needs of diverse communities and stakeholders, the WIN process tried to ensure a balanced approach to selecting measures in multiple dimensions. This approach balanced leading indicators and reported outcome measures with a low measurement collection burden that could move quickly and be used for real‐time improvement by people in health care, social services, business, and many community‐based organizations. The WIN process also used lagging indicators that moved more slowly over time to meet the needs of public health, housing and community development, planners, and other governmental stakeholders. An effort was made to include a mix of upstream, midstream, and downstream indicators, data from both national measures and subcounty measures, and outcome measures reported by people as well as more standard secondary measures. Equity was prioritized in both the process and the outcomes during the selection of measures.
**5. A focus on creating a living library of measures**. Such a library could evolve with learning about what works instead of “a definitive set” of measures “for all time.” Besides the identification of core measures and leading indicators, WIN introduced a “flexible” set of innovative measures and a process through which they could be rigorously evaluated. Recognizing the need to adapt to special populations, WIN created the space and formed a process for enabling different expert groups to lead. All this made it possible for many groups to choose to “WIN” together.

### Work Groups

In mid‐2017, 100 Million Healthier Lives convened three work groups—metrics development, measurement implementation, and stewardship—to further develop the NCVHS framework and make recommendations regarding the objectives and scope of the proposed measurement ecosystem. The work groups recommended including two additional domains: equity and the well‐being of people. A subset of the stewardship group in collaboration with the NCVHS Population Health Subcommittee's members then conducted an initial landscape analysis of candidate measures for inclusion in the framework.

### Modified Delphi Cycles

The modified Delphi Cycle participants represented all domains of the NCVHS framework. In all, 108 individuals from 69 federal and nonfederal organizations contributed to the process. Eighteen communities nationwide helped test the measures for utility, and approximately 25 organizations working with aligned measurement efforts contributed their input. The measures being considered were evaluated according to modified National Quality Forum (NQF) criteria, such as importance, objectivity and effectiveness, feasibility, and usability and use.^13^ The stewardship group, whose members represented multiple sectors, including employers, health and health care, and media, contributed details to the National Quality Forum decision criteria (Box 1).^14^

**Box 1:** Decision Criteria, Adapted From the National Quality Forum Criteria for Evaluating a Measure

**Overall (basic information for all nominated metrics)** Domain Subdomain Proposed metric Source of metric Link to website for more information Level of data available (national, state, county, subcounty, zip code, community, etc.)
**Important** Potential to improve health Potential to improve social drivers of well‐being Potential to improve equity Aligned with major national/global strategy Potential to develop new knowledge about what creates well‐being **Objective and effective** Strong evidence that this improves health, well‐being, and equity Valid Reliable Benchmarking available
**Feasible** Data already collected, analyzed, and reported Cost of additional collection/availability of resources to support collection Burden of collection and reporting Groups ready to adopt
**Usable and useful** Time‐frame data changes (rating: 3 if less than quarterly, 2 if less than yearly, 1 if yearly, 0 if more than yearly) Timeliness of data availability (rating: 3 if less than quarterly, 2 if less than yearly, 1 if yearly, 0 if more than yearly) Usefulness to communities Usefulness to researchers/national stakeholders Meaningfulness to people with lived experience Currently used by/could be used by (name initiatives, organizations actively using) Level of data availability

The WIN measures were selected using five cycles of a modified Delphi Process (Table 1), Through this process, more than 100 groups and local communities worked together to put hundreds of measures into a living library of measures that could be used across sectors.

**Table 1:** Well‐Being in the Nation (WIN) Process

	Process	Output
Landscape analysis	Identification of both measurement efforts and measures.	500+ measures and 50+ measurement efforts and implementation efforts identified.
Engagement	Leads from major measurement efforts and implementation efforts identified. Formation of Stewardship Group, Measure Development, and Measure Implementation groups.	100+ organizations and communities engaged.
Delphi Cycle 1: Identification of missing candidate measures	Participants suggested additions to the list of candidate measures, derived from their expertise or familiarity.	Complete list of candidate measures generated.
Delphi Cycle 2: Prioritization of candidate measures	In each domain, participants prioritized 10 measures for inclusion in each of the national and community measure sets based on the measure's importance, value/usefulness, and usability to stakeholders.	Approximately 20 of the most selected measures per domain at each national and community level.
Delphi Cycle 3: Evaluation of candidate measures	In each domain, participants prioritized five measures for inclusion in each of the national and community measure sets and then evaluated the measures’ importance, feasibility, usability, and value on a scale of 1 (least) to 3 (most).	Parsimonious set of measures at national and community levels.
Delphi Cycle 4: Expert validation of candidate measures	Two to six experts in each domain/sector of the framework evaluated Cycle 3 outputs. Measures were then categorized into Leading Indicators and Flexible Expanded Set based on importance and data availability.	Modified parsimonious set of measures: Core Measures, Leading Indicators, and Flexible Expanded Set.
Delphi Cycle 5: Alignment of measures with existing initiatives	Outputs of the expert validation cycle (Cycle 4) were compared with measures used in other major initiatives and reviewed with implementers. The major gaps and alignment opportunities were also addressed.	Refined Core Measures, Leading Indicators, and Flexible Expanded Set integrated into existing initiatives and measurement efforts.
Formation of WIN Network and “Living Library” process	Core body of implementers engaged to actively lead implementation to learn together and make WIN a “living library of measures” that is refined as the field learns together.	Collaborative WIN network chosen by implementers to advance implementation, refine the measures with added testing, and add policy and narrative strategies.

## Findings

“Well‐being in the Nation (WIN) Framework: Measures for Improving Health, Well‐Being and Equity Across Sectors” was published as a report in June 2019 together with a website developed at a ninth‐grade reading level with core measure and leading indicator data equitably available to communities. Measures in WIN are organized into core measures, leading indicators, and a flexible expanded set of measures (Table 2), which can be found at www.winmeasures.org.

**Table 2:** Well‐Being in the Nation (WIN) Measures

	Description	Measure Organization	Example
**Core Measures**	Nine measures to be used across initiatives.	**Well‐being of people** People reported well‐being Life expectancy **Well‐being of places** Child poverty Healthy community indices aligned with the framework **Equity** Differences in well‐being Years of life lost Income inequality High school graduation rates Demographic variables to use in a standard way for equity analysis	**Well‐being of People** Cantril's ladder: Please imagine a ladder with steps numbered from 0 at the bottom to 10 at the top. The top of the ladder represents the best possible life for you, and the bottom of the ladder represents the worst possible life for you. Where would you put yourself now? In 5 years?**Well‐being of places** County Health Rankings & Roadmaps USNWR Healthiest Communities Rankings **Equity**Race, place, education, language, sexual identity, veteran status, disability, urban/rural
**Leading Indicators**	Highly recommended measures related to determinants of health and well‐being of people, places, and equity that have readily available data.	Community vitality Economy Education Environment and infrastructure Equity Food and agriculture Health Housing Public safety Transportation Well‐being Demographics	Social‐emotional support Perception of racial inclusion Unemployment rates Availability and quality of affordable housing Availability and quality of healthful food Self‐perceived health Deaths of despair Juvenile incarceration Availability of transportation
**Flexible Expanded Set**	Highly promising, valid measures that require additional adoption and research or lack data availability; promising as a future leading indicator.	Organized in same categories as leading indicators.	Everyday Discrimination Scale Sense of purpose and meaning Social isolation

*Core Measures* (*N* = 9) are grouped into three themes:

1. *Well‐being of people*. The well‐being of people is measured by their perception of their own well‐being (using Cantril's ladder) and their life expectancy at birth.

Used most frequently in the business sector as well as by Gallup‐Healthways and the RWJF Culture of Health Survey, Cantril's ladder^15^ is a highly validated, two‐question item, publicly available, person‐reported outcome measure used in the Gallup World Poll:

Imagine a ladder with steps numbered from zero at the bottom to ten at the top. The top of the ladder represents the *best possible life for you* and the bottom of the ladder represents the *worst possible life for you*. 1. Indicate where on the ladder you feel you personally stand right now. 2. On which step do you think you will stand about five years from now?

Cantril's ladder has been in use since 1965, and has been administered more than 2.7 million times through Gallup National and Well‐Being Index assessments in hundreds of communities across the United States and in more than 150 countries around the world with data available at the subcounty level and in multiple demographic fields. Using only two questions that tested as both very easy to administer across communities with a wide range of literacy levels and contexts, the score was movable in testing in local communities within 6 to 12 months.^16^ This mattered to local communities, health care systems, and payers because it was easy to see improvements or setbacks in the percentage of people suffering (on average, the top 3.2% of the population at highest risk), struggling (medium/rising risk for 39.5% of the population), and thriving (57.3% of the population).^17^ These categories correlated with morbidity, mortality, cost, and worker productivity in a way that was important for driving meaningful improvement in health, well‐being, and societal outcomes.^18^, ^19^ Cantril's ladder was also an international standard, one of two measures recommended by the Organisation for Economic Co‐operation and Development (OECD) to measure population health and well‐being.^20^

Life expectancy at birth was chosen as a second measure for the well‐being of people. This highly validated measure is used widely in population health rankings. While it is a lagging indicator, the recent availability of data at a Census tract level has made it an important measure associated with both the health of groups of people and as a marker of place‐based equity.

2. *Well‐being of places*. The well‐being of places is measured by the child poverty rate as a single measure or by one of two indices of healthy communities aligned with the domains and subdomains of the NCVHS framework, the US News & World Report Healthiest Communities Rankings,^21^ and the County Health Rankings & Roadmaps.^22^

These indices include community‐level measures from the leading indicators in domains such as the built environment, community vitality and belonging, transportation, and the economy, and offer a high‐level view of a community's structures and systems.

Child poverty was chosen as a single indicator after multiple measurement groups independently identified it as their preferred single indicator of a community's health and well‐being because it correlates with a number of other community‐based indicators, like long‐term child development and population health outcomes.^23^

3. *Equity*. Equity is measured by the differences in perception of well‐being and premature death (years of life lost), income inequality, and high school graduation rates based on demographic factors such as race, place (zip code), gender, language, and urban/rural/suburban.

In addition to the Core Measures, the WIN framework offers the following:

- A parsimonious set of “Leading Indicators” aligned with the NCVHS domains of community vitality, economy, education, environment and infrastructure, food and agriculture, health, housing, public safety, transportation, and demographics (measures for the well‐being of people and equity domains fall under “Core Measures” and “Flexible Expanded Set”). These measures have been well tested, have data available at the county or subcounty level, and can be benchmarked.
- A flexible, expanded set of highly recommended measures (Flexible Expanded Set) aligned with all WIN domains, including established and innovative measures for every domain and subdomain across the life course. This set includes measures with early evidence of significant impact, for example, a person's perception of everyday discrimination, a sense of purpose and meaning in life, and measures of community vitality, social isolation, and belonging.

NCVHS reviewed and supported both the process and the outcome of the WIN measure development process.

## How the WIN Measures Are Being Used

The best measure of a framework's value is its adoption in the field. By its launch time, through the process of sharing the emerging WIN framework with those who had participated in its development process, more than 15 national implementers reaching hundreds of communities across the country had adopted the WIN framework and integrated the core measures and leading indicators in their work (See Box 2). In addition, over the next several months, more than 50 organizations reaching thousands of communities, including both federal and state agencies, also adopted these measures. Healthy People 2030 has intentionally aligned with many of WIN's core measures and leading indicators. The Centers for Medicare and Medicaid Services is also considering using some of WIN's core measures for health care system accountability. The Administration for Community Living funded the National Councils on Aging to test use of WIN core measures of well‐being with older adults. Their use in Baltimore led to policy and funding changes to support expansion of senior center hours and supports for older adults. Because WIN built on what communities already valued, hundreds of communities that are already using County Health Rankings & Roadmaps to guide progress, can continue to do so with additional enhancements as needed. WIN measures serve as the foundation for the Springboard for Equitable Recovery and Resilience post‐COVID.

**Box 2:** Ways That WIN Measures Are Being Used in Communities

Downtown Women's Center (DWC), the first housing services provider dedicated to serving women on skid row in Los Angeles, has a small clinic dedicated to meeting the health needs of women experiencing homelessness. The center adapted the Diabetes Prevention Program in partnership with these women and used a combination of clinical measures (A1C, BMI, blood pressure) and WIN measures to evaluate its progress. Within six months, compared with a control group and controlling for housing placement, the center observed that 30% more women were thriving and fewer were suffering, with demonstrated accompanying improvements in clinical outcomes. In addition to a number of small clinics like the DWC, a number of large health systems, such as Kaiser Permanente, Health Partners, Providence St. Joseph, Methodist Healthcare Ministries, and Adventist, have adopted the WIN measures to assess their impact on their patients and the community. In Delaware, the Division of Substance Abuse and Mental Health (DSAMH) has convened a multisector collaborative across state agencies (police, corrections, social service, foster care, etc.) and community providers (emergency rooms, addiction providers, hospitals, etc.) to meet the needs of people with addictions. DSAMH is using a combination of WIN measures such as overall well‐being, deaths of despair, years of life lost/gained, employment, housing, other social needs met, and legal issues resolved to support people in real time and to focus and evaluate their impact across sectors. In Fox Cities, Wisconsin, community leaders across sectors came together to look at intergenerational well‐being, community vitality, and basic needs. Because they had stratified their data and used powerful measures that everyone could understand, they learned that 80% to 92% of their communities of color were struggling or suffering. This discovery led to communitywide dialogues about inclusion as well as consideration of policy and system changes to support racial and economic inclusion.

The WIN measures are being used for the following:

1. To identify measures for national initiatives that can be applied across a wide variety of communities (eg, the American Heart Association).
2. To monitor the health, well‐being, and equity of a population over time (assessments by numerous communities, counties, states, and the nation).
3. To understand and drive improvement in health, social needs, and social determinants in organizations and communities across sectors by using the relevant measures before, during, and after their implementation.
4. To evaluate population health and social determinant initiatives at regional and state levels.
5. To conduct research studies that connect the impacts of different interventions on well‐being.
6. To understand the health, well‐being, and equity in population segments, such as older adults, veterans, and the corrections population.
7. To compare the health and well‐being of communities through the development of an index and rankings.

The groups and communities that participated in the WIN measurement framework development work decided to form a “WIN measurement cooperative” to continue to update the living library of measures as they discover and learn during implementation. They are working together to collectively inform federal, state, and local data strategies to create an equitable measurement system that supports the needs of local communities. In addition, a WIN network has been formed across organizations to shift narratives, policies, and systems in a way that would drive improvement in the measures.

### Delaware COVID‐19 Tracking Case Study

In the context of COVID‐19, the Delaware Division of Substance Abuse and Mental Health (DSAMH) was able to rapidly deploy WIN measures to assess and address the well‐being of people with mental health and addictions. Using a set of linked Excel files, DSAMH rapidly deployed a daily tracker and engaged hundreds of care managers to proactively reach out to people with mental health and addictions. In addition to a general check‐in, care managers assessed people for COVID‐19 symptoms, mental health symptoms, and five questions related to self‐perceived overall well‐being, hope for the future, financial insecurity, loneliness, peer support, housing, and legal needs. These questions were also integrated into incoming calls and in‐person encounters.

In Delaware, the average population baseline of people suffering, based on Cantril's ladder, is about 3.5%. In the first measurement period between March 16 and March 27, 2020, DSAMH noted that suffering using Cantril's ladder was 15%. Thirty‐eight percent of people were lacking hope that things would improve in five years. This was alarming because responses in the suffering range are correlated with morbidity, mortality, and disease, and lack of hope that things will improve in the long term is highly correlated with deaths of despair. These results seemed to correlate with people's financial insecurity, with 40% reporting that they were suffering from financial insecurity and 26% reporting a lack of social support. To date, with 8,465 well‐being assessments collected, financial well‐being and social connectedness seem to be highly correlated with overall well‐being outcomes (*r* = 0.431, *p* < 0.0001 and *r* = 0.528, *p* < 0.0001, respectively).

DSAMH mobilized a rapid response to address social needs such as housing and food and to connect people with financial resources, as well as online and telephonic peer supports in response to these results, training all care managers to use Cantril's ladder to stratify and support patients in real time. They also made sure that people who did not show up for care were followed up with and that those who had COVID‐19 symptoms were connected to primary care and those who had acute mental health needs were connected to the right care providers.

Over the next two months, DSAMH watched the percentage of people suffering rise to a high of 25% and then come back down again to 5%. This almost certainly resulted from a combination of policies at the federal, state, and local level, including stimulus funding for people who were unemployed. For DSAMH, the ability to create a real‐time well‐being monitoring system for its population was useful as a barometer and a guide for where to focus.

## Discussion

The WIN framework aims to meet the needs of stakeholders that are struggling to improve population health and to address social determinants and equity across sectors. The framework's early adoption in the field has been the best indicator of its value to stakeholders and reflects their engagement in the framework's development. Furthermore, it fulfills the intentions of Public Health 3.0, which recommends that

timely, reliable, granular‐level (i.e., sub‐county), and actionable data should be made accessible to communities throughout the country, and clear metrics to document success in public health practice should be developed … a core set of metrics that encompass health care and public health, particularly the social determinants of health, environmental outcomes, and health disparities.^24^

As these measures become increasingly integrated into communities’ and states’ assessments of needs, national initiatives, and research efforts, the WIN collaborative will continue to convene people across stakeholders and local communities. The WIN framework is designed to evolve over time as users collectively learn which drivers and measures are predictive of the key outcomes of improving the well‐being of people, the well‐being of places, and equity.

WIN was faithful to the intentions and goals of the NCVHS framework by advancing the committee's work toward implementation. The first phase of WIN was an inclusive, public‐private effort to transform the vision of a federal advisory committee—with its detailed needs assessment and measurement framework containing recommendations for the HHS secretary—into a methodical stakeholder engagement process that produced a publicly available resource with specific national, county, and subcounty community measures across sectors.

WIN supports the intentions of the framework for the Evidence‐Based Policymaking Act and related Federal Data Strategy goals, which require that federal “agencies will identify an initial set of priority agency datasets that are key to mission success and/or a priority for stakeholders outside of the agency” and to “facilitate data sharing between state, local, and tribal governments and the Federal Government, where relevant and appropriate and with proper protections … to enable richer analyses for more informed decision‐making.”^25^ The first‐year action plan of the Federal Data Strategy (FDS) is largely focused on the infrastructure to govern and manage federal data assets. In response to the FDS's request for comment on the action plan, NCVHS recommended that the FDS look beyond this initial plan: “It is vitally important that the FDS prioritize the development of a process for producing publicly consumable health information from federal health data assets.”^26^

Most communities across the nation do not have the resources to identify and test relevant measures or to collect enough broad‐based data to support decision making. By identifying the essential measures for use at the state and local levels, WIN will be able to provide a blueprint for federal data producers that, over time, could result in greater data quality and accessibility at a lower cost to federal, state, county, and local governments. Another result of the WIN framework and measures will be the ability of communities to benchmark and compare outcomes, enhancing cross‐community collaboration and learning for improving health, well‐being, and equity. This is a concept that was launched within HHS by the Community Health Status Indicators (CHSI) in 2000 using county‐level data but has recently been discontinued.^27^

## Policy Implications

WIN has made available a well‐vetted and well‐used set of measures that simultaneously serve local, state, and federal goals for evidence‐based action and health equity. The service of this public‐private partnership to the Federal Data Strategy and to HHS's Public Health 3.0 goals cannot be overstated. Nonetheless, the opportunity of this convergence will not be fully realized unless it is recognized by the Federal Data Strategy (FDS). The FDS's intentions of “leveraging data as a strategic asset” will fall short if the FDS does not consider strategy when attending to the needs of stakeholders.^28^ WIN offers a clear, flexible menu of measures to support population health that has been chosen by more than 100 organizations and communities, many of which are now using the measures to drive improvement. These measures need to be reliably available to local (subcounty) and state stakeholders through federal data assets.

The standardization of these measures does not preclude local innovations. In the meantime, these measures create a common playing field for assessment, planning, action, and evaluation, greatly enhancing multisector collaboration. WIN's measures could serve as a foundation for a coordinated national approach to measuring and developing shared strategies across sectors for improving individual and community health and well‐being.

The WIN Network and 100 Million Healthier Lives have made WIN measures publicly available at the subcounty level through a public‐private partnership with LiveStories. In the future, the data for common measures like the WIN measures that are valued by communities across the country should be prioritized and funded to be equitably available to communities through the new Federal Statistical Research Data Centers (FSRDCs) that have been created to house these data. In a joint statement advising the Federal Data Strategy, more than 40 WIN partners representing local communities, major national organizations, and federal measurement processes recommended the following policies be implemented to ensure communities’ equitable access to data:

1. Federal data, gathered at taxpayers’ expense, need to be publicly available and accessible in an equitable way to local communities and analyzed at the subcounty level.
2. The input of communities and other cross‐sector stakeholders, as well as federal agencies, should drive national priorities in the Federal Data Strategy.
  - This includes determining what data are accessible to support measurement, based on a process that has received substantial input from communities and nonfederal stakeholders, such as the Well‐Being in the Nation measurement framework (which contains data related to the County Health Rankings & Roadmaps and the US News and World Report Healthiest Community Rankings).
  - This includes where analytic capability is focused to collect and make data available at the local (subcounty) level.
  - This includes how data are made available for sociodemographic subpopulations to ensure that we can understand disproportionate harm as well as opportunities.
  - This includes how data are made available at the local level to support national objectives, such as those captured in Healthy People 2030.
3. The identification of data priorities should be based on fair and equitable processes, and ideally a public‐private partnership, such as the one conducted by NCVHS to identify these priorities.
4. The availability of data and capacity in order for local communities to use the data will best be achieved through public‐private partnerships in collaboration with federal agencies.
5. Steps should be taken to ensure that the data cannot be used to target a population or individuals in any way that would harm their well‐being.

The nation is just beginning to learn how to connect the dots and set policy together across the drivers of well‐being—health, economy, housing, education, and civic life—in a way that works for the diversity of communities in more than 3,000 counties, is equitably accessible, and offers a way to learn what works. The COVID‐19 crisis has made apparent the connections among these spheres. Perhaps even more important than offering a set of common measures that communities can use as a start, the process of developing WIN measures offers a model that can be used to advance a multisector learning measurement system to support a learning health and well‐being system that connects the community level to the national level.

*Funding/Support*: Support for this work came from multiple sources through both financial and in‐kind resources (eg, staff time, meeting hosting). In‐kind support was provided by the Institute for Healthcare Improvement (convening partner for 100MLives, provided staffing support for facilitation of the development process), LiveStories (related to WIN website development), members of the NCVHS Committee, and all the experts who participated in the Delphi process and work groups. Resources to support gatherings were provided by the Well Being Trust, Kaiser Permanente, and the Institute for Healthcare Improvement. Finally, support to ensure that data would be available to communities through an interactive website was provided by the Robert Wood Johnson Foundation and the Well Being Trust.

*Acknowledgments*: The authors would like to acknowledge Rebecca Hines at the Centers for Disease Control who served as our liaison with the National Committee on Vital and Health Statistics, as well as Carley Riley at Cincinnati Children's and Brita Roy at Yale New Haven University for their invaluable support as part of the design team for the Well‐being In the Nation (WIN) measure development process.

*Conflict of Interest Disclosures*: None.
